# Sex‐Specific Factors Influencing HIV Infection

**DOI:** 10.1111/imr.70109

**Published:** 2026-02-20

**Authors:** Maria Pujantell, Marcus Altfeld

**Affiliations:** ^1^ Department Virus Immunology Leibniz Institute of Virology (LIV) Hamburg Germany; ^2^ Institute of Immunology University Medical Center Hamburg Eppendorf (UKE) Hamburg Germany

**Keywords:** escape from XCI, HIV‐1, IFN pathway, immunity, RNA sensing pathway, sex differences, TNF pathway, X‐chromosome

## Abstract

Immune responses are key in controlling viral infections such as HIV‐1, which remains a global challenge to public health. X/Y‐chromosome‐encoded genes and sex steroid hormones can modulate immune cells and drive distinct patterns of gene and protein expression involved in antiviral function, impacting sex‐dependent immune responses and altering the balance of specific immune pathways. These sex‐specific differences in antiviral immune responses have significant consequences for the outcome of HIV‐1 infection. A better understanding of sex differences in HIV‐1‐specific antiviral immunity is required to implement and develop new strategies for prevention, treatment and ultimately a functional cure against HIV‐1. Here we review the impact of X‐ and Y‐encoded genes and the role of sex steroid hormones on modulating antiviral immune responses against HIV‐1 and the consequences for disease manifestations in people living with HIV‐1.

## 1 Sex, Gender and HIV

Biological sex influences the risk, progression, and treatment of many diseases due to genetic, hormonal, and physiological differences between males and females [1, 2]. Several studies have shown that females tend to mount stronger innate and adaptive immune responses against pathogens compared to males [1, 3]. However, enhanced immune responses in females come at a cost, as females display higher immune activation, persistent inflammation, and are more susceptible to autoimmune diseases compared to males [4]. In the context of studies investigating sex‐specific differences in diseases, it is important to distinguish between gender and biological sex, as both have implications for human health and access to healthcare. Gender refers to social, cultural, personal choices, and constructs encompassing behavior, identities, and social roles that may differ from biological sex and can independently influence health through social expectations, behaviors, and access to care [2, 5]. In this review, we will focus on biological mechanisms mediated by sex steroid hormones and genes encoded by the X and Y chromosome that impact immunity.

Human immunodeficiency virus 1 (HIV‐1) can cause the acquired immune deficiency syndrome (AIDS) if left untreated. Thanks to the introduction of combination antiretroviral therapy (cART), HIV‐1 infection has become a manageable chronic disease. HIV‐1 integrates into the host DNA and full elimination of the virus is currently not possible, except in very exceptional cases in which a cure has been achieved through bone marrow transplantations [6, 7, 8, 9, 10, 11, 12]. In addition to access to cART, efforts to control the HIV‐1 pandemic depend heavily on coordinated local, governmental, and intergovernmental actions. Policy changes, available funding, stigma and unequal access to healthcare can all directly affect HIV‐1 transmission and control. By the end of 2024, UNAIDS estimated 40.8 million people living with HIV (PLWH) worldwide and 1.3 million new infections in 2024. However, recent shifts in funding priorities, public health policies and HIV‐1 prevention strategies are risking long‐standing efforts to control the HIV‐1 epidemic, already resulting in increased numbers of new infections [13, 14, 15].

HIV‐1 is a retrovirus that primarily infects T lymphocytes through the CD4 receptor and co‐receptors CXCR4 and/or CCR5, but can also infect and persist in other immune cells, such as macrophages and dendritic cells. In infected cells, HIV‐1 establishes a persistent latent viral reservoir by integrating into host DNA. During acute infection, pathogen‐associated molecular patterns (PAMPs) present in HIV‐1 are sensed by pathogen‐recognition receptors (PRRs). This activates antiviral signaling cascades that induce transcription of interferon‐stimulated genes (ISGs), trigger release of different cytokines and chemokines, and recruit other immune cells, initiating an antiviral innate and adaptive immune response against HIV‐1. In combination, these antiviral responses aim to restrict viral replication, alert neighboring cells, and ultimately limit virus spread. However, HIV‐1 has evolved multiple mechanisms to evade and suppress antiviral immune responses, enabling the establishment of a persisting viral infection and preventing viral elimination.

Although the immune systems of males and females perform the same principal immune functions against pathogens, sex‐specific differences in antiviral immune responses are present due to differential expression of X‐ and Y‐chromosomal genes and variations in hormone‐dependent immune modulation. As a result, significant sex‐specific differences are observed in HIV‐1 susceptibility, disease progression, and treatment outcomes. In general, PBMCs derived from women have higher proportions of total T cells, especially naïve CD4^+^ T cells, and B cells, while PBMCs derived from men have a higher frequency of monocytes and NK cells and particularly higher percentages of regulatory T cells (Treg) [16, 17]. During primary HIV‐1 infection, women present with lower HIV‐1 viral loads, higher CD4^+^ T cell counts, and higher CD4:CD8 ratios than men [18]. Women living with HIV‐1 (WLWH) however tend to have higher levels of chronic immune activation and faster decline of CD4^+^ T cells than men living with HIV‐1 (MLWH), after controlling for levels of viral replication [19, 20, 21]. Therefore, despite lower initial HIV‐1 viral loads, WLWH develop AIDS at similar rates as MLWH. Epidemiologically, cis‐gender females have higher susceptibility to HIV‐1 acquisition during heterosexual transmission, due to a combination of anatomical, hormonal, and microbiological factors [22]. For instance, mucosal inflammation in the female genital tract is known to lower the barrier for HIV‐1 infection due to tissue remodeling and enhanced availability of activated CD4^+^ target cells [23], promoting HIV‐1 acquisition. During intrauterine HIV‐1 transmission, female infants also have a higher risk than male infants to acquire HIV‐1 [24], and females overall have a 1.7‐fold increased risk of perinatal acquisition than males, even in the presence of antiretroviral therapy [25, 26, 27]. In addition, viruses transmitted to female infants are more likely to be interferon‐resistant [24], while male infants exhibit lower TLR7/8 responses [28], suggesting a sex‐specific selective pressure during perinatal infection [27, 29]. Other immune cells involved in HIV‐1 pathogenesis, such as female macrophages, are reported to exhibit higher phagocytic activity than male macrophages [1, 18] and display higher activation levels compared to male macrophages. In this case, sex‐dependent differences in SAMHD1 phosphorylation have been shown to drive SAMHD1 activity and macrophage activation levels, influencing susceptibility to HIV‐1 infection [30]. These findings highlight how subtle changes in phosphorylation levels can alter activation and phagocytic capacity, resulting in sex‐specific differences in HIV‐1 susceptibility.

Importantly, significant differences in the production of cytokines that play a critical role in HIV‐1 control and HIV‐1‐associated pathology have been reported between males and females. A now well‐established pathway that is regulated by sex‐specific factors is the TLR7‐pathway in pDCs. Female pDCs exhibit enhanced IFNα production compared to male pDCs after stimulation with TLR7 ligands, such as ssRNA derived from HIV‐1 [28, 31, 32, 33, 34, 35, 36], and it has been shown that genes encoded by the X chromosome and sex steroid hormones can directly regulate the TLR7 pathway. Furthermore, in the context of other infections such as HCMV, it has been shown that T cells from females produced higher levels of IFNγ and IL‐2 than T cells from males [37, 38], suggesting that multiple immune pathways leading to cytokine production in response to viral infections can be regulated in a sex‐specific manner. HIV‐1 elite controllers are a small subset (< 1%) of PLWH that are able to maintain undetectable or very low levels of HIV‐1 replication in the absence of cART. WLWH are over‐proportionally represented in cohorts of HIV‐1 elite controllers, consistent with the described superior ability of controlling viral replication.

HIV‐1 infections result in the establishment of a persistent latent viral reservoir that represents one of the major challenges for HIV‐1 cure efforts, and several mechanisms have been suggested to regulate HIV‐1 latency in a sex‐specific manner. One of the best investigated mechanisms is the role of the estrogen receptor 1 (ERα) that contributes to latency by repressing HIV‐1 promoter activity and transcription through inhibiting a complex between ERα and β‐catenin [18, 23, 39, 40]. Most studies report comparable HIV‐1 reservoir sizes between WLWH and MLWH [41, 42, 43], although a few studies have reported lower levels of HIV‐1 DNA in women [44, 45]. However, more recent studies using next‐generation techniques for reservoir quantification (reviewed here [46]) are now providing more detailed insights into the complex characteristics of HIV‐1 reservoir heterogeneity and size, integration sites, and gene expression profiles that can depend on the sex of the host cells. Recent data from single genome amplification in peripheral blood suggest an intriguing sex‐specific structure and compositions of provirus [47]. Female cells infected with HIV‐1 were observed to have higher frequencies of genome‐intact provirus integrated in heterochromatin‐rich regions compared to male cells. This observation is important, as these heterochromatin regions represent sites that are transcriptionally repressed due to dense chromatin structure, potentially enabling a better control of latency of HIV‐1 proviruses [47]. In line with the more frequent integration of HIV‐1 provirus in heterochromatin‐rich regions in female cells is the observation that WLWH have lower levels of multiply spliced cell‐associated HIV‐1 RNA and lower residual viremia than men, suggesting tighter innate immune control of viral transcription post‐integration [18, 22, 48]. In addition, epigenetic regulatory factors display sex differences and can contribute to the observed sex‐specific differences in HIV‐1 latency. Women exhibit in general higher levels of DNA methylation in studies using whole blood [49]. Although DNA methylation does not directly predict gene expression, it is considered to take part as a broader regulatory network of gene control [50]. The specific drivers of sex‐biased DNA methylation patterns in female whole blood are not fully understood. However, evidence from the brain tissue, where sex differences in DNA methylation have been most studied, shows that these patterns are established early during development and persist to adulthood [51, 52]. Contributing factors to sex‐biased DNA methylation patterns may include X‐linked epigenetic modulation, such as KDM6A [53, 54], as well as sex‐hormone epigenetic modulation [49, 55]. However, further research is needed to decipher the relative contribution of these mechanisms to the overall epigenetic regulation and their consequences for control of HIV‐1.

Taken together, while there is an increasing consideration of sex as an important biological variable in HIV‐1 research, there is still insufficient data available on sex‐specific factors that modulate HIV‐1 infection and pathogenesis, in part due to an unequal representation of sex and gender in published studies. In this review, we will focus on the role of genes encoded by the X and Y chromosome, as well as the role of sex steroid hormones, in regulating HIV‐1 pathogenesis.

## 2 Role of Genes Encoded By Sex Chromosomes In HIV‐1 Infection

The specific contributions of genes encoded by the X‐ or Y‐chromosome to sex differences in the manifestations of HIV‐1 infection remain insufficiently understood. The principal factors that might contribute to sex differences reviewed in this article are the escape of X‐chromosomal genes from the inactivation of the second X chromosome (X chromosomal inactivation, XCI) in female cells, the functional specificity of Y‐chromosome‐encoded homologous proteins in male cells, and the impact of Y‐chromosome haplogroups.

### 2.1 Consequence of XX genotype in females

Because female cells have two X chromosomes, one X chromosome is inactivated (XCI) to balance X‐encoded gene dosage. However, some genes on the inactive X chromosome can escape XCI and hence are expressed from both X chromosomes, resulting in higher gene and protein expression of these genes. This includes enhanced gene expression of immune‐related genes that can augment antiviral immunity in female cells. Different reviews have already discussed the implications of escape from XCI for immune function [56, 57, 58, 59, 60, 61]. Here, we will focus on selected X‐chromosome‐encoded genes that are involved and/or can potentially modulate immune responses to HIV‐1. Although additional X‐chromosomal genes might have a direct or indirect role in HIV‐1 immune responses, their direct influence has not yet been well established, and these genes were therefore not included in this review.

#### 2.1.1 X‐Encoded Genes Regulating the Type‐I IFN Signaling Pathway

##### 2.1.1.1 TLR7

Toll‐like receptor 7 (TLR7) is part of the Toll‐like receptor (TLR) family, which are type I transmembrane receptors that recognize PAMPs expressed by foreign pathogens. TLR7 recognizes particularly single‐stranded RNAs (ssRNA) that contain guanosine‐ and uridine‐rich sequences from RNA viruses. TLR7 proteins are expressed in the endosomes of immune cells such as pDCs, NK cells, and B cells. It has been shown by several studies that TLR7 signaling contributes to sex differences in immune responses due to its role in regulating type‐I IFN responses. Sex‐specific differences in TLR7 responses have been reported in several studies for pDCs. TLR7 has been shown to escape from XCI [34, 36], leading to biallelic expression in some XX cells. The higher TLR7 gene and protein expression [28, 36, 62] in female pDCs with biallelic TLR7 expression resulted in increased type‐I IFN responses after TLR7 stimulation compared to female monoallelic and male pDCs. The enhanced response through TLR7 signaling enhances downstream signaling pathways through MyD88‐dependent activation of IRF7 and NF‐kB, which amplifies type‐I IFN production and other pro‐inflammatory cytokines. Stronger TLR7 signaling pathways and type‐I IFN responses in pDCs might therefore contribute to the observed female bias in antiviral immunity. The importance of TLR7 and stronger TLR7 signaling for HIV‐1 disease outcome has been highlighted by studies of SNP haplotypic markers in the *TLR7* gene. *TLR7* rs179008 c.32 T allele carriers have been shown to have decreased production of IFN‐α by pDCs in WLWH on cART [63]. Chronic HIV‐1‐infected individuals furthermore displayed enhanced TLR7 function due to higher mRNA transcription after TLR7 stimulation [63]. In addition, females carrying the rs179008 AA SNPs in TLR7 exhibited enhanced TLR7 function [63]. In a study of only cis‐gender WLWH on cART, the magnitude of type‐I IFNs after TLR7 stimulation was inversely associated with the size of the latent HIV‐1 reservoir [64], suggesting that stronger type‐I IFN responses contribute to lower HIV‐1 reservoir sizes in WLWH [64]. In addition, studies of vertical HIV‐1 transmission have shown that female sex represented a risk factor for in utero HIV‐1 acquisition and was linked to the transmission of interferon‐resistant HIV‐1 strains with higher viral replication capacity than those transmitted in utero to males [24, 29]. Interestingly, cord blood cells from female newborns of HIV‐1‐uninfected sex‐discordant twins were more strongly activated and more susceptible to HIV‐1 in vitro than cells from male newborns, suggesting that intrinsic host factors can drive sex differences in susceptibility to HIV‐1 early in life [24].

Activation of TLR7 through TLR7 agonists has furthermore demonstrated the capacity to induce activation of latent HIV‐1 reservoirs in CD4^+^ T cells, while at the same time activating antiviral T and NK cell responses [65, 66], representing a potentially promising immune‐therapeutic strategy to reduce the size of latent HIV‐1 reservoirs in PLWH. Clinical data from the HIVACAT T‐cell Immunogen (HTI) therapeutic vaccine [67], which is designed to elicit T‐cell responses against conserved HIV‐1 Gag, Pol, Vif, and Nef protein fragments in PLWH, further showed that when combined with a TLR7 agonist, the vaccine had enhanced immunogenic and antiviral effects during analytical treatment interruption [67]. However, the study only included male participants, and additional studies in WLWH and MLWH are required to fully elucidate the potential therapeutic benefits for PLWH. Mechanistically, the modulation of the HIV‐1 reservoir through TLR7 signaling has been shown to mainly occur in memory CD4 T cells, where upon activation of the TLR7‐IRF5 axis memory CD4 T cells are sensitized to Fas‐mediated apoptosis, providing a potential mechanism by which TLR7 agonists contribute to reservoir depletion [68]. Taking together, sex‐specific differences in TLR7 expression and function have been shown to impact antiviral Type‐I IFN responses, and their therapeutic potential for HIV‐1 cure approaches is currently investigated.

##### 2.1.1.2 TLR8

Similar to TLR7, Toll‐like receptor 8 (TLR8) recognizes PAMPs expressed by infectious agents and induces the production of cytokines necessary for an innate immune response. TLR8 specifically senses G‐rich oligonucleotides, and the TLR8 homodimer has two binding sites that recognize two degradation products of ssRNA, uridine and a short oligonucleotide that stabilizes the TLR8 dimer complex necessary for downstream signaling [69, 70]. TLR8 is located in endosomes, recognizes ssRNA from different viruses, including HIV‐1 and HCV, and triggers a signaling cascade similar to TLR7, mediated by MyD88 and nuclear translocation of transcription factors NF‐kB, MAPK/AP‐1, and IRFs, resulting in the production of different proinflammatory cytokines and Type‐I IFNs. TLR8 is not expressed by pDCs but by T cells, and has been shown to activate T cells by strengthening the TCR signaling pathway, while similar to TLR7, furthermore reversing HIV‐1 latency in infected CD4^+^ T cells derived from PLWH [71, 72, 73, 74]. In latently infected CD4 T cells, TLR8 stimulation leads to the activation of NF‐κB signaling that reactivates HIV‐1 transcription and also induces the production of TNFα that activates neighboring latently HIV‐1‐infected CD4 T cells [72]. Emerging evidence furthermore points to synergistic interactions between TLR signaling and RIG‐I‐like receptors (RLR). Cross‐activation of TLR with RLR and latency reversal agents (LRA) enhanced antiviral immunity and induced antiviral immune response mediated by Type‐I IFN responses that can reduce the size of the HIV‐1‐inducible reservoir [75, 76]. Suggesting that TLR8 stimulation can both “shock” the latent virus by inducing reactivation through the activation of the innate immunity, and also “kill” infected cells in combination with LRAs makes it a potential candidate in strategies aimed at reducing the HIV‐1 reservoir. Similar to TLR7, the TLR8 signaling cascade works in coordination with other X‐chromosome–encoded proteins such as IRAK1 and TASL, suggesting that in conjunction these signaling molecules encoded by the X‐chromosome can modulate HIV‐1 replication and also reactivation.

##### 2.1.1.3 IRAK1

Interleukin‐1 receptor‐associated kinase 1 (IRAK1) is a key signaling molecule in the downstream TLR pathway, specifically TLR7 and TLR8. In the context of HIV‐1, IRAK1 has therefore emerged as an important regulator of innate immune activation and inflammatory signaling. In particular, escape from XCI of IRAK1 might result in higher IRAK1 expression in female cells and contribute to the described higher Type‐I IFN responses to HIV‐1 in female cells. Inhibition of IRAK1 can block pro‐inflammatory cytokine production upon TLR8 signaling by both synthetic ligands and HIV‐1 derived RNAs [77]. This suggests that chronic innate activation could be reduced by reducing IRAK1 activity and inhibition of downstream pro‐inflammatory signaling, suggesting IRAK1 as a potential target to reduce immune activation in HIV‐1 infection, including neuro‐inflammation [77]. Therefore, a better understanding of the sex‐dependent effects of IRAK1 may offer new strategies to reduce chronic inflammation in HIV‐1 and improve personalized clinical outcomes.

##### 2.1.1.4 TASL/CXorf21

TLR adaptor interacting with SLC15A4 on the lysosome (TASL), also known as CXorf21, is an immune regulatory protein that functions as an essential adaptor in TLR signaling. TASL interacts with SLC15A4 and is required for the activation of IRF5 downstream of TLR7, TLR8 and TLR9, leading to induction of Type‐I interferon and pro‐inflammatory cytokines [78, 79]. Through modulation of this pathway, TASL contributes to innate immune sensing of viral nucleic acids. In mice, the *Tasl* gene has been shown to escape X‐chromosomal inactivation in immune cells [80]. In humans, TASL exhibits female‐biased expression, which has been associated with increased susceptibility to autoimmune diseases and is consistent with expression patterns of other X‐linked immune genes reported to escape XCI. However, direct evidence of TASL escape from XCI has not yet been reported in pDCs [81, 82]. Increased TASL gene dosages have been linked to enhanced TLR7‐IRF5 signaling and elevated Type‐I IFN‐responses, a mechanism implicated in sex‐biased immune activation and susceptibility to autoimmune disease such as systemic lupus erythematosus [81]. These findings support TASL as a contributor to sex differences in innate immune signaling through X‐chromosome gene‐dosage effects. Direct evidence linking sex‐biased TASL expression to HIV‐1 transcription, replication, or latency is currently lacking. However, TASL regulates TLR7/8 signaling pathways and IRF5‐dependent IFN‐responses, which are important during HIV‐1 infection and contribute to immune activation and inflammation, suggesting a potential role in antiviral responses to HIV‐1. Taken together, TASL represents an X‐linked innate immune regulator whose sex‐biased expression may influence antiviral and inflammatory signaling and contribute to sex differences in immune responses during HIV‐1 infection.

##### 2.1.1.5 DDX3X

DDX3X is a member of the DEAD‐box RNA helicase family and a protein with many potential functions that might play a role during different viral infections. In HIV‐1 infections, DDX3X has been shown to interact with viral RNA structures and proteins to regulate different processes such as transcription, RNA export, translation, and virion assembly (reviewed [83]). DDX3X has been reported to interact with Gag, Rev., Tat and Vpu/Vpx [84, 85], as well as RNA elements such as the TAT hairpin and Rev. Response Element (RRE). Through these interactions, DDX3X has been suggested to enhance viral gene expression and replication during HIV‐1 infection while also antagonizing IFN‐I signaling to impair innate immune responses through RIG‐I, MAVS and TBK1 [84, 86, 87]. DDX3X inhibitors have therefore been proposed as a novel class of host‐directed antiviral agents, holding promise for PLWH infected with multidrug‐resistant HIV‐1 strains or being co‐infected with HCV [88]. Recent studies have shown that DDX3 is expressed at higher levels in pDCs derived from females, potentially due to escape from XCI, and that it can promote higher levels of IFNα production after TLR7 stimulation in female pDCs [89], indicating a potential contribution to sex differences in innate immune responses to HIV‐1. Further research is required to better understand how escape of DDX3 from XCI might impact other aspects of viral replication in a sex‐specific manner, and whether DDX3 activity is influenced by hormonal regulation.

#### 2.1.2 X‐Encoded Genes Regulating T Cell Functions

##### 2.1.2.1 FOXP3

FOXP3 is a transcription factor encoded by the X‐chromosome that regulates the development and function of regulatory T cells (reviewed [90]). FOXP3 suppresses immune responses by activating genes that promote T regulatory cell phenotype and repressing genes that induce inflammatory responses, given that mutations in the FOXP3 gene cause autoimmune disorders such as IPEX (Immune dysregulation, Polyendocrinopathy, Enteropathy, X‐linked) syndrome. FOXP3 expression is regulated by different pathways, including IL‐2 signaling, TCR/CD28 co‐stimulation, and TGF‐β receptor signaling [91]. In addition, sex‐specific factors might modulate FOXP3 expression and regulatory T cell function, with testosterone and other androgen‐receptor agonists enhancing FOXP3 expression [91, 92]. As a result, cis‐gender males display higher FOXP3 mRNA levels in both CD4^+^ and CD8^+^ T cells compared to cis‐gender females [91, 92], despite FOXP3 being encoded by the X chromosome. The involvement of FOXP3 expression in different T cell subsets and its association to HIV‐1 disease progression has been more complex to decipher. FOXP3^+^CD8^+^ T cells have been associated with HIV‐1 disease progression [93], and elevated levels of FOXP3 double negative (DN) T cells have been consistently observed in untreated PLWH with low CD4 T cell counts [94]. DN FOXP3 T cells negatively correlate with CD4^+^ T cell count and CD4:CD8 ratio, and positively correlate with immune activation and systemic inflammation in PLWH [94], linking this T cell subset to progressive HIV‐1 disease. Similarly, expansion of FOXP3^+^CD4^+^NKT‐like cells has also been associated with HIV‐1 disease progression in PLWH with enhanced immune activation and systemic inflammation, and PLWH with higher baseline levels of FOXP3^+^CD4^+^NKT‐like cells were more at risk of poor immune reconstitution after initiation of cART [95]. In addition, epigenetic regulation of the FOXP3 promoter site has been shown to be significantly demethylated in PBMCs and colon mucosa of PLWH [96], suggesting enhanced FOXP3 transcription activity due to HIV‐1. Taken together, these data suggest that sex‐dependent modulation of FOXP3 expression, influenced by hormonal and epigenetic mechanisms, might contribute to differential expansion of FOXP3+ T cell subsets during HIV‐1 infection. Interestingly, despite its location on the X chromosome, FOXP3 expression appears to be higher in males, resulting from enhanced expression in response to androgen receptor activation.

##### 2.1.2.2 CXCR3

CXCR3 is a chemokine receptor mostly expressed on CD4^+^ and CD8^+^ T cells that is potentially involved in the pathogenesis of HIV‐1 through several functions, such as trafficking, activation, and differentiation of lymphocyte subsets. CXCR3 binding to the chemokine IP‐10 (CXCL10) has been shown to enhance latent HIV‐1 infection in resting CD4^+^ T cells by promoting activation and actin remodeling that facilitate viral entry and integration [97]. In chronic HIV‐1 infection, CXCR3 and CXCR5 are highly expressed on HIV‐1‐specific central memory CD8^+^ T cells [98], suggesting a role for CXCR3 in regulating migratory and functional profiles associated with antiviral surveillance. Dysregulation of CXCR3 expression has been furthermore linked to delayed CD4^+^ T cell reconstitution in immunological nonresponders that exhibited elevated proportions of CXCR3+ CCR6‐ Th1‐like circulating Tfh cells. These cells correlated negatively with CD4^+^T cell numbers, suggesting that altered Tfh polarization might be regulated by CXCR3 and contribute to poor immune T cell reconstitution [99]. On the other hand, CXCR3^+^ T follicular helper cells and CXCR3^+^ B cells in lymph nodes positively correlate with the development of HIV‐1‐specific antibody responses in acute HIV‐1 infection, indicating that CXCR3 might also contribute to the induction of germinal center responses and subsequent antibody production [100, 101]. Finally, a distinctive memory‐like NK cell population characterized by expression of CD16, NKG2A, NKG2C, CD57, and CXCR3 has been described in HIV‐1 elite controllers that displayed increased cell metabolism, migration, and antiviral effector functions [102]. These findings suggest expression of the CXCR3 receptor as a shared immunological feature by different immune subsets in the context of HIV‐1 infection.

A sex‐specific regulation of CXCR3 expression has been suggested by several studies. Although not in the context of HIV‐1, a mouse model of *Leishmania mexicana* infection showed that biallelic CXCR3‐expressing T cells produce higher levels of CXCR3 protein than monoallelic CXCR3‐expressing T cells [103]. Furthermore, biallelic CXCR3‐expressing T cells produced higher levels of IFN‐γ, IL‐2, and CD69 compared with monoallelic CXCR3‐expressing T cells [103]. These findings suggest that higher levels of CXCR3 contribute to enhanced T cell activation and cytokine production, suggesting improved effector immune responses in this infection model. Taken together, given that CXCR3 expression and downstream signaling can be influenced by sex‐specific factors, these sex differences may affect both the magnitude and dynamics of CXCR3 expression on different immune cell subtypes, including memory subsets, with potential consequences for antiviral T cell immunity and antibody responses against HIV‐1.

##### 2.1.2.3 XIAP/xIAP

The X‐linked inhibitor of apoptosis protein (XIAP or xIAP) regulates T cell activation, survival and susceptibility to apoptosis. XIAP expression is enhanced after CD4^+^ and CD8^+^ T cell activation, and functional studies have demonstrated that siRNA‐mediated XIAP knockdown reduces T cell activation [104]. Importantly, higher XIAP expression has been observed in PLWH that experience rapid disease progression, and it has been suggested that XIAP supports HIV‐1 viral replication by delaying CD4^+^ T cell apoptosis [104]. Antiapoptotic effects and cell survival supported by XIAP might also contribute to long‐term persistence of HIV‐1 by enhancing survival of latently HIV‐1‐infected cells [104, 105]. In line with the anti‐apoptotic role of XIAP in infected CD4^+^ T cells, blocking of XIAP enhanced the elimination of HIV‐1‐infected cells [106, 107]. These data suggest that XIAP, and other cellular survival factors, contribute to viral persistence and the maintenance of HIV‐1 reservoir. Future studies are required to understand whether the location of XIAP on the X‐chromosome results in sex‐specific expression or effects of XIAP on HIV‐1 reservoir sizes and viral persistence in PLWH.

##### 2.1.2.4 KDM6A/UTX

KDM6A, also known as UTX, is a histone demethylase encoded by the X chromosome that catalyzes the removal of di‐ and tri‐methyl groups from histone H3 lysine 27 (H3K27me2/3), promoting chromatin accessibility and transcriptional activation [108, 109, 110]. UTX has a homolog on the Y chromosome named UTY (KDM6C) that has been reported to also have catalytic function (catalytic demethylation of H3K27) but at a lower level than KDM6A/B [111]. UTX has been shown to escape from XCI in female cells, and might therefore regulate HIV‐1 replication and antiviral immunity in a sex‐specific manner. In the context of HIV‐1 replication, UTX has emerged as a potentially critical epigenetic regulator of viral transcription and latency [112, 113]. UTX has been shown to facilitate HIV‐1 gene expression through modulation of chromatin at the HIV‐1 LTR [112, 113]. UTX can regulate Tat‐driven transactivation by downregulating the repressive H3K27 methylation mark and promoting the accumulation of the activating H3K4 methylation mark at the HIV‐1 LTR, thereby enhancing HIV‐1 transcription. Mechanistically, demethylation of H3K27 enhances chromatin permissibility that directly allowed increased nuclear translocation of NF‐κB p65 and binding of p65 to HIV‐1 LTR, promoting transcription of the LTR and enhanced HIV‐1 replication [112]. Modulation of UTX has therefore been proposed as a strategy to maintain provirus silencing of HIV, as pharmacological inhibition of UTX supports H3K27 methylation, enhancing proviral repression and inhibiting spontaneous reactivation [113]. However, UTX inhibition alone was not sufficient to completely block HIV‐1 reactivation upon strong immune activation [113], suggesting that a combination with other histone modulating factors might be necessary for latency control.

UTX has also been shown to escape from X‐chromosomal inactivation in NK and T cells of females [53, 114, 115]. In both cell types, UTX regulates chromatin accessibility, inducing transcription at different loci and modulating a network of genes involved in effector function, frequency, and durability of virus‐specific immune cells [53, 114]. Mechanistically, higher UTX expression in female cells enhances transcription at the *Ifng, Gzmb*, and *Prf1* loci, which correlates with enhanced effector function in female NK cells [53]. In a similar pattern, UTX binds to enhancers and transcription start sites of effector genes in T cells, promoting robust early CTL‐mediated antiviral protection in CD8^+^ T cells [114]. At the same time, UTX limits the frequency and durability of virus‐specific CD8^+^ T cells through enhanced expression of inhibitory receptors and reduced long‐term persistence of CD8^+^ T cell responses [114]. UTX might therefore function as a double‐edged sword, facilitating HIV‐1 proviral transcription by enhancing chromatin accessibility, while simultaneously promoting the antiviral effector function of cytotoxic immune cells targeting virus‐infected cells [53, 114].

### 2.2 Consequences of XY genotype in males

In contrast to the X chromosome, the contribution of the Y chromosome to HIV‐1 pathogenesis remains poorly understood. This knowledge gap is partly due to the fact that Y‐linked genes are present exclusively in males and lack allelic compensation, resulting in male‐specific gene dosage effects. In addition, many transcriptomic and genetic studies in the past have excluded the Y chromosome or lack sufficient resolution to capture Y‐linked regulatory effects, limiting insights into Y chromosome‐mediated male‐specific effects in HIV‐1 infection and pathogenesis. Here, we review the few recent insights into the role of the Y chromosome and its genes that might have implications for HIV‐1 disease outcome.

#### 2.2.1 Y‐Chromosome Haplogroups

Genetic studies have suggested that variation in the Y chromosome, captured by Y‐chromosome haplogroups, might contribute to variability in HIV‐1 disease outcomes. Y‐chromosome haplogroups have been associated with susceptibility to HIV‐1 infection and differences in HIV‐1 disease progression in infected men [116, 117]. In particular, Y‐chromosome haplogroup I (hg‐I) in MLWH has been associated with more rapid progression to AIDS and AIDS‐related death, delayed viral suppression on HAART, and a higher frequency of AIDS‐defining illnesses [116]. These associations persisted after controlling for known autosomal genetic factors, suggesting that Y‐linked genetic variation may influence HIV‐1 pathogenesis. Although Y‐chromosome haplogroups themselves are not functional, their association with HIV‐1 outcomes suggests underlying Y‐linked loci that modulate immune or inflammatory mechanisms, including protein coding and noncoding RNAs.

#### 2.2.2 NonCoding RNA (ncRNAs) Encoded by Y Chromosome

The human Y chromosome is one of the smallest human chromosomes and contains the fewest number of protein‐coding genes compared to the autosomes [118, 119]. Despite the limited amount of genes, approximately 40% of the Y chromosome consists of euchromatin [118], indicating active transcription and suggesting that the Y chromosome has further regulatory function after early sex determination [118]. Indeed, the Y chromosome encodes many noncoding RNAs (ncRNAs), including long noncoding RNAs (lncRNAs), microRNAs (miRNAs), and circular RNAs (circRNAs). Multiple linear *lncRNAs* are encoded by the *Y chromosome*, but less is known about the function of Y‐linked lncRNAs compared to lncRNAs encoded by autosomes (reviewed [118]). Several studies have linked autosomal lncRNAs to HIV‐1 replication and pathogenesis (reviewed [120]). lncRNAs have been detected in key HIV‐1 target cells including CD4^+^ T cells, monocytes and macrophages, where they regulate immune activation and viral gene expression [121, 122, 123]. In contrast, no Y‐linked lncRNAs have been described to date that play a role in HIV‐1 replication, latency, reservoir formation and persistence, or antiviral immune responses. Consequently, Y‐linked lncRNAs represent an unexplored regulatory layer that may contribute to male‐specific immune responses during HIV‐1 infection. Defining the functions of Y‐encoded ncRNAs will be essential to fully understand sex chromosome contribution to HIV‐1 disease and may uncover novel mechanisms underlying sex differences in antiviral immunity.

#### 2.2.3 UTY/KDM6C

The UTY protein is encoded by the *KDM6C* gene and represents the paralog of the X‐linked UTX (KDM6A) gene expressed in immune cells (reviewed [124]). While UTX functions as an active H3K27me3 demethylase that induces transcription activation, UTY displays reduced catalytic activity due to alterations in its catalytic domain [111]. Nevertheless, UTY maintains its chromatin‐binding capacity and is involved in transcription regulation, even though it has no enzymatic function. Y‐linked chromatin regulators, including UTY, have been shown to modulate autosomal gene transcription with genome‐wide regulatory effects [125, 126]. In male cells, UTY and UTX are co‐expressed, whereas female cells contain two copies of UTX, resulting in sex‐specific differences in the balance of H3K27 methylation patterns and epigenetic differences [127]. HIV‐1 latency and reactivation are tightly regulated by chromatin structure such as H3K27me3 at the viral promoter, and modulation of these marks might therefore strongly affect proviral transcription [128]. Although UTY has not been directly linked to HIV‐1 replication or latency establishment, male‐specific differences in epigenetic regulation mediated by UTY could influence HIV‐1 transcriptional control and therefore deserve future attention.

#### 2.2.4 KDM5D/SMCY/JARID1D

The lysine‐specific demethylase 5D (KDM5D) protein (also known as SMCY/JARID1D) is encoded by the *KDM5D* gene on the Y‐chromosome. KDM5D is a histone demethylase that removes activating H3K4me3 and H3K4me2 marks, inducing transcription repression [129] (reviewed [124]). KDM5D is the paralog of X‐linked KDM5C and retains catalytic activity, participating in chromatin remodeling and transcriptional regulation [130] (reviewed [124]). As HIV‐1 transcription and latency are influenced by H3K4 methylation at the HIV‐1 promoter, KDM5D could therefore also have implications for HIV‐1 latency and reactivation. However, no data are available on sex differences in KDM5D expression and their effects on HIV‐1 pathogenesis, and future studies in this area are required.

#### 2.2.5 Mosaic Loss of Y‐Chromosomes (mLOY)

Several studies have described that the Y chromosome can be lost in male somatic cells, a process termed mosaic loss of Y (mLOY). mLOY is more frequent in older men, as the Y chromosome is more prone to mitotic loss and has been associated with immune aging [131, 132, 133, 134]. More recent studies have shown that mLOY is not uniform across tissues and is associated with altered transcriptional landscapes, immune dysfunction, and increased disease susceptibility [125, 134, 135]. Importantly, mLOY has been observed in CD4^+^ and CD8^+^ T cells, where it is associated with transcriptional dysregulation and impaired immune responses [136]. These findings provide important evidence that Y‐linked genes contribute to immune regulation in males and raise the possibility that mLOY could influence HIV‐1 disease progression, immune activation and reservoir dynamics in males. However, the impact of mLOY on HIV‐1 disease has not yet been directly studied.

Taken together, very few studies have addressed the role of genes on the Y chromosome in regulating viral replication and antiviral immunity, including HIV‐1. This area clearly represents an understudied area that requires future investigations.

## 3 Role of Immune Regulation By Sex Steroid Hormones In HIV‐1 Infection

Hormones are signaling molecules produced by the endocrine system, and sex steroid hormones can regulate several physiological processes, such as sexual development, reproductive functions, and secondary sexual characteristics, but also metabolism and immune function. Although females and males produce the same type of hormones, steroid hormones significantly differ in concentrations and temporal patterns of hormone production between the sexes, with estrogens, progesterone, and androgens representing the principal sex steroid hormones in the blood. These steroid hormones exert their function through binding to specific receptors, commonly classified as cell membrane receptors and nuclear receptors. Cell membrane receptors, located on the target‐cell membrane, transduce extracellular signals into rapid intracellular responses, whereas nuclear receptors act as transcriptional regulators. Nuclear receptors bind hormone response elements (HREs) within target gene promoters, with high specificity determined by nucleotide repeats recognized by the receptor DNA‐binding domain [137, 138]. In addition to direct DNA binding, nuclear hormone receptors act as cofactors by binding to other transcription factors, such as NF‐kB/AP1, modulating transcription and epigenetic patterns [139, 140].

Sex steroid hormonal levels change across the lifespan. Infancy is characterized by growth hormone‐driven development, followed by a surge of estrogen and testosterone during puberty that induces the development of secondary sex characteristics and the release of hormones from the hypothalamus (GnRH) and the anterior pituitary (LH and FSH). During reproductive age, females experience cyclic fluctuations of estrogen and progesterone during the menstrual cycle, as well as high hormone levels during pregnancy and lactation. Males exhibit peak testosterone levels following puberty that gradually decline with age. Later in life, sex steroid hormones decline in both sexes. While female menopause is characterized by a more rapid drop in estrogen and progesterone levels, male menopause is associated with a more gradual androgen decline. Additional steroid hormones such as the adrenal hormones (DHEA) and growth hormones also decline with age. Overall, these sex‐ and age‐differences in sex steroid hormone levels impact gene expression and function of immune cells through receptor signaling, further modulating pre‐existing sex‐specific epigenetic patterns in blood and tissues. PLWH can experience gonadal and hormonal changes and dysfunctions, which have been best characterized in women [141]. These HIV‐1 ‐associated variations in sex steroid hormones add an additional layer of complexity to the studies of sex‐differences in HIV‐1 pathogenesis.

### 3.1 Estrogen‐Mediated Effects in HIV‐1 Pathogenesis

Estrogens are important regulators of immune function and have been extensively studied in HIV‐1 pathogenesis. Estrogens are derived from androgens and are present in multiple biologically active forms. 17‐β Estradiol (E2) is the most potent estrogen, while other estrogens play different roles at specific life stages, such as estriol (E3) and estetrol (E4) during pregnancy [142], estrone (E1) representing the predominant postmenopausal estrogen produced mainly by adipose tissue and adrenal glands [143], and 27‐hydroxycholesterol (27HC), a cholesterol‐driven estrogenic metabolite maintained at low circulating levels [144, 145].

Estrogen signaling is mediated by estrogen receptor alpha (ERα) encoded by the *ESR1* gene, estrogen receptor beta (ERβ) encoded by the *ESR2* gene, and G‐protein coupled receptor 30 (GPR30 or GPER1) encoded by the *GPER1* gene. Both ERα and ERβ have multiple spliced variants that might encode functional proteins with different affinities for estrogens, although their function remains incompletely characterized [146, 147]. Quantitative gene expression analyses showed distinct receptor distribution across immune cell subsets: *ESR1* is highly expressed in B cells, moderately expressed in CD4^+^ T cells, CD8^+^ T cells, NK cells, and pDCs, and expressed at low levels in monocytes [33]. *ESR1* expression is however increased in monocyte‐derived DC, suggesting a role in DC development [33]. In contrast, *ESR2* expression is high in B cells and pDCs, while low in CD4^+^ T cells, CD8^+^ T cells, monocytes and NK cells, and very low in monocyte‐derived DCs [33]. GPER1 expression is highest in naïve B cells, γδ T cells, CD8^+^ T cells and activated memory CD4^+^ T cells, with lower expression in memory B cells, macrophages, resting memory CD4^+^ T cells and Tregs [148]. Upon estrogen‐binding, GPER1 mediates rapid non‐genomic signaling, including intracellular calcium mobilization [149], MAPK pathway activation [150], and cAMP production [149, 151]. In contrast, ERα activation leads to nuclear translocation and transcriptional regulation through estrogen response elements (ERE) or via interaction with transcription factors such as RUNX1 [152], AP‐1 [153], and Sp1 [154]. Taken together, these heterogeneous distributions, distinct expression levels and differential signaling pathways result in very complex effects of estrogens and their metabolites on immune functions that are only insufficiently understood, in particular in vivo.

In the context of HIV‐1 pathogenesis, estradiol has been shown to inhibit HIV‐1 replication in PBMCs [155, 156, 157]. Mechanistically, E2 suppresses HIV‐1 LTR transcription by preventing binding of β‐catenin and ERα to the HIV‐1 promoter [155]. In addition, hormone concentration represents a critical determinant of estrogenic effects on HIV. Estradiol and progesterone levels corresponding to the mid‐proliferative phase of the menstrual cycle (lower concentrations) are associated with increased HIV‐1 transcription, whereas higher concentrations typical of the mid‐secretory phase reduce viral integration [156]. However, the precise contribution of this mechanism to a superior control of HIV‐1 viremia in WLWH remains unclear, as sex differences in HIV‐1 control are already apparent before puberty, with female children exhibiting lower HIV‐1 RNA levels and higher CD4 percentages than male children living with HIV‐1, despite similar levels of T cell activation [158]. Estrogen furthermore directly modulates innate immune responses relevant to control of HIV‐1. Estradiol regulates IRF5 expression in pDC, with higher IRF5 levels observed in females that positively correlate with IFN‐α production [159]. Furthermore, reduction of estrogen levels in trans‐gender men has been associated with reduced IFN‐α production by pDCs [160, 161]; it remains however unknown whether this reduction in IFN‐α production is a consequence of reduced estrogen levels or elevated levels of testosterone. In addition to these direct and immune‐mediated effects of estrogens on HIV‐1 replication, estrogen signaling has also been suggested to influence HIV‐1 latency and persistence. Estradiol can inhibit latency reversal and proviral reactivation of HIV‐1 in CD4^+^ T cells, with stronger inhibition observed in cells from females compared to males [162]. In the same study, WLWH exhibited a smaller total inducible HIV‐1 RNA reservoir than MLWH [162]. Longitudinal HIV‐1 reservoir analysis further reveals sex‐specific differences in reservoir dynamics: total HIV‐1 DNA declined more slowly in WLWH on cART, while the transcriptionally active HIV‐1 RNA+ reservoir increases in WLWH after menopause, suggesting a potential role for estrogen withdrawal in reservoir reactivation [163]. Taken together, the results from these studies indicate that estrogen levels represent an important variable in HIV‐1 replication, persistence and immune control, and should be included in the analysis of HIV‐1 disease outcomes in cis‐ and trans‐gender PLWH.

### 3.2 Progesterone‐mediated effects in HIV‐1 pathogenesis

Progesterone (P4) is best known for its role in pregnancy in women; however, it also plays important roles in male physiology. Progesterone concentrations vary throughout life and are generally higher in women than in men. Progesterone signaling is mediated by progesterone receptors (PRs), which are expressed in many reproductive and nonreproductive tissues [164]. Two major classes of PR have been described: membrane‐bound PRs (mPR) and nuclear PR (nPR). mPR signaling is transduced through G‐protein activation and MAPKs [165], cAMP/PKA signaling, and other downstream pathways (reviewed [166]). In contrast, nPR exists as two isoforms: PR‐A and PR‐B, and the relative expression of PR‐A versus PR‐B contributes to distinct biological activities of P4 [167]. nPRs are expressed by many immune cells [168]. The PR‐B isoform localizes to both the cytoplasm and nucleus and has been shown to exert extranuclear signaling, whereas the PR‐A isoform is predominantly nuclear and mediates transcriptional regulation [169, 170]. Nuclear progesterone signaling starts by ligand binding to nPR, which subsequently binds to progesterone response elements (PREs) to regulate gene transcription.

Studies investigating the effect of progesterone on HIV‐1 infection and pathogenesis are limited, but progesterone levels have been suggested to be associated with increased susceptibility to HIV‐1 in some studies. Several studies suggested that progesterone can enhance HIV‐1 acquisition risk in highly exposed women largely based on epidemiological evidence from studies investigating the impact of hormonal contraceptive, in particular progesterone‐rich DMPA, that was linked to enhanced HIV‐1 acquisition risk [156, 171]. However, it remains unknown whether the enhanced risk for HIV‐1 acquisition was primarily due to the effects of progesterone, or rather due to distinct risk behavior and risk exposures between women using progesterone‐containing injectable contraceptives or oral or no hormonal contraceptives. Mechanistically, progesterone can induce the upregulation of HIV‐1 entry receptors CD4, CCR5, and CXCR4 on human cervical CD4^+^ T cells [172, 173, 174, 175]. Progesterone has furthermore been suggested to enhance HIV‐1 acquisition risk [176] through upregulation of inflammatory cytokines and chemokines and downregulation of anti‐apoptotic pathways, enhancing HIV‐1 replication in infected cells [176]. The progesterone‐high luteal phase of the menstrual cycle might therefore create a potential window of vulnerability for HIV‐1 acquisition, and in vitro studies using cervical tissue explants have shown increased susceptibility to HIV‐1 infection during that phase [177, 178]. Innate, humoral, and cell‐mediated immune responses are also more suppressed during mid‐cycle to facilitate the possible implantation of the fertilized egg for successful initiation of pregnancy [179], potentially further facilitating viral transmission and HIV‐1 acquisition during that phase. Taken together, progesterone signaling adds an additional layer of hormonal regulation that modulates antiviral immune function and susceptibility to HIV‐1 acquisition in females of reproductive age in which progesterone levels fluctuate during the menstrual cycle.

### 3.3 Androgen‐mediated effects in HIV‐1 pathogenesis

Androgens are the predominant male sex hormones, but they are also present in women at lower levels than men. Androgens are a family of steroid hormones, including the testosterone precursors dehydroepiandrosterone (DHEA), DHEA sulfate (DHEAS) and androstenedione that can be converted into the highly potent androgen metabolites testosterone and dihydrotestosterone (DHT), and also estrogen [180]. Androgen signaling is mediated through the androgen receptor (AR), a nuclear hormone receptor encoded by the X‐chromosome and expressed by multiple immune cell types, including T cells, B cells, macrophages, neutrophils and mast cells [181, 182, 183]. Upon ligand binding, ARs translocate to the nucleus and regulate gene transcription by binding to androgen response elements (ARES) or by interacting with other transcription factors, including NF‐κB and AP‐1, modulating the expression of many immune‐related genes [184, 185]. Several studies in mice have identified sex‐biased immune cell distributions and gene expression patterns that were modulated by androgens [186, 187, 188]. These studies in mice have been more recently complemented by studies in humans undergoing gender‐affirming hormone therapy (GAHT) with androgens. Androgen‐based GAHT was shown to induce a shift in immune cell profiles in trans‐gender men that closely resembled those observed in cis‐gender men. In addition, androgen‐based GAHT impacted the function of immune cells in trans‐gender men. For example, IFNα‐responses of pDCs and also monocytes in response to TLR7‐stimulation were downregulated in trans‐gender men compared to levels observed prior to initiation of GAHT levels [160, 161]. At the same time, androgen‐based GAHAT increased NF‐κB signaling in monocytes, including the production of TNF, IL‐6 and IL‐15 after TLR4 stimulation, promoting downstream IFN‐γ responses by NK cells [161].

Androgen levels in men decline with age, but this decline has been described to be accelerated in MLWH. Multiple studies have shown reduced testosterone levels in MLWH, leading to a high prevalence of hypogonadism, estimated to range from 13% to 40% depending on age and disease state, including cART‐treated men [189, 190, 191, 192, 193, 194, 195]. HIV‐1 infection has furthermore been described to alter androgen levels and sex hormone‐binding globulin (SHBG) concentrations in WLWH, leading to reduced circulating androgen and estradiol levels accompanied by elevated SHBG levels, resulting in decreased bioavailable sex steroid hormones compared to women without HIV‐1 [196, 197, 198]. The precise consequences of these alterations in bioavailable hormone levels for antiviral immunity and HIV‐1 pathogenesis remain incompletely understood. However, it has been described in multiple studies that androgens display immunosuppressive effects, including reduced T cell activation and inflammatory cytokine production [199], suggesting that androgen signaling may influence HIV‐1 pathogenesis by modulating T cell activation thresholds, inflammatory cytokine production, and immune exhaustion, rather than directly regulating viral transcription [184, 200]. Taken together, androgens may modulate HIV‐1 pathogenesis through immunosuppressive effects rather than directly affecting HIV‐1 replication or latency, as suggested for estrogens. However, the mechanistic link between androgens and HIV‐1 replication and pathogenesis is less well understood. Addressing the gap will require mechanistic studies investigating the relationship between hormone levels, immune cell compositions and function, as well as HIV‐1 replication to determine how androgen signaling contributes to sex‐specific differences in antiviral immune responses and clinical outcomes in HIV‐1 infection.

## 4 Concluding Remarks

**FIGURE 1:**
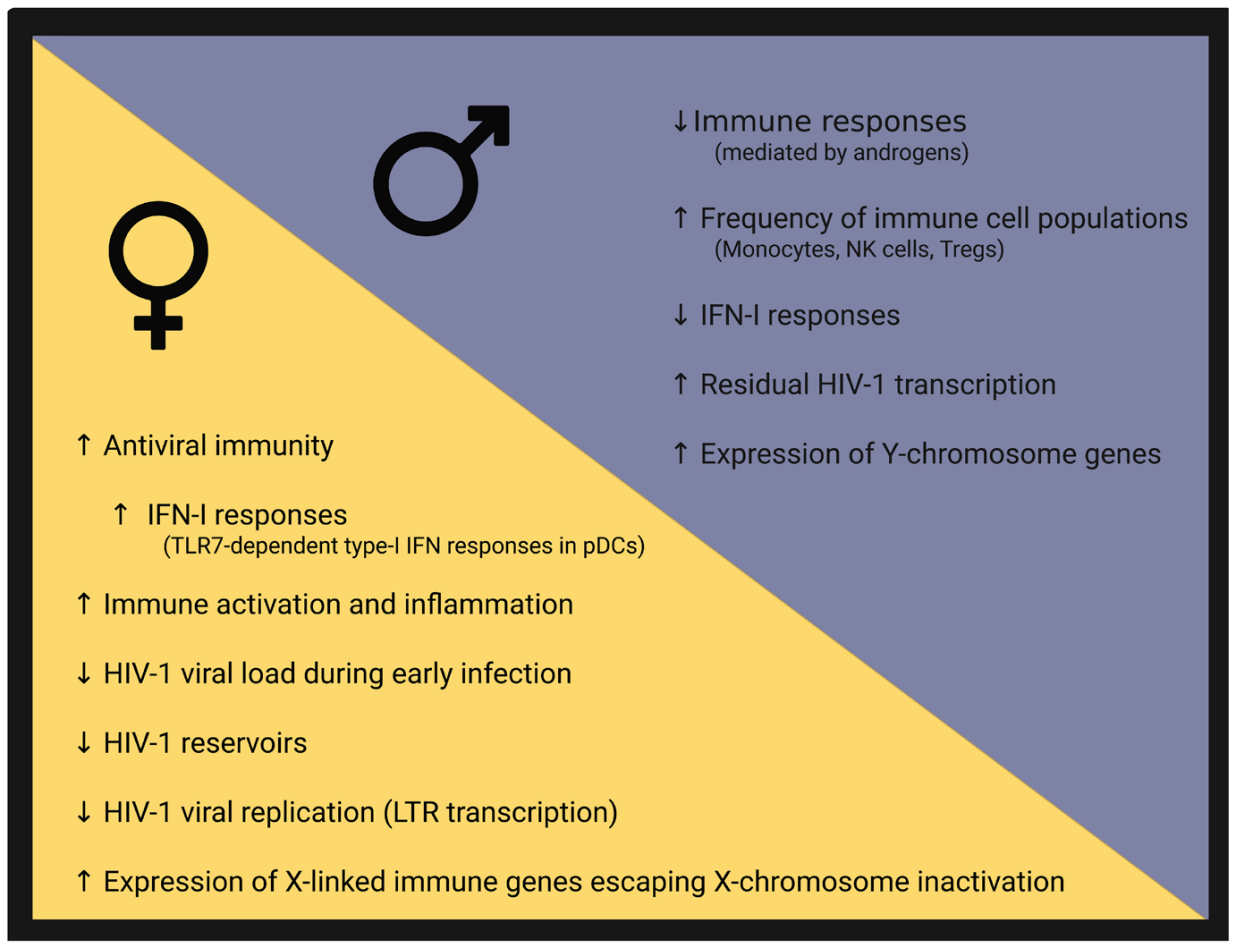
**Sex differences in HIV‐1 disease**. Key biological sex‐dependent effects resulting from the influence of sex chromosomes and of sex hormones are shown. Predominant immune‐mediated effects observed in females are demonstrated on the left (yellow), including enhanced antiviral immunity, type‐I IFN responses and inflammation that are associated with reduced early viral replication and HIV‐1 reservoir sizes. Predominant immune‐mediated effects observed in males are shown on the right (blue), including weaker antiviral immune responses resulting from androgen signaling that are associated with higher residual HIV‐1 transcription. Arrows indicate relative increase (↑) or decrease (↓).

Biological sex influences HIV‐1 susceptibility, disease progression and viral persistence through the combined effects of sex chromosome‐encoded genes and sex hormones on immune function (Figure 1). Females generally mount stronger antiviral immune responses, characterized by enhanced innate immune sensing, especially the TLR7‐dependent Type‐I IFN responses in pDCs. In HIV‐1 infection, this enhanced antiviral immunity is associated with lower viral loads during early infection, higher CD4^+^ T cell counts and CD4:CD8 ratios, and enhanced immune activation and inflammation in WLWH. Female sex is furthermore linked to superior control of viral persistence, including estrogen‐mediated repression of HIV‐1 transcription, lower residual viremia and cell‐associated HIV‐1 RNA on cART (Figure 1). In contrast, males exhibit immune profiles driven by androgen signaling, with higher frequencies of monocytes, NK cells and regulatory T cells, enhanced FOXP3 expression, weaker TLR7‐dependent Type‐I IFN responses, and higher levels of residual HIV‐1 transcription and viremia. Androgen‐mediated immunosuppression can furthermore dampen T‐cell activation and inflammatory responses. Together, these sex‐specific effects reflect a balance between antiviral control and immune activation that shapes the course of HIV‐1 pathogenesis and viral persistence, with important implications for the design of cure strategies (Figure 1).

## Funding

This work was supported by Deutsche Forschungsgemeinschaft (DFG), Research Unit 5068‐Sex Differences In Immunity.

## Conflicts of Interest

The authors declare no conflicts of interest.

## Data Availability

This study is a review of previously published literature. All data supporting the findings of this study are available within the cited articles.
